# Evaluation of the Cytotoxic Effect of Calcium Silicate-Based Bioceramic Sealers: An In Vitro Comparative Study

**DOI:** 10.7759/cureus.116891

**Published:** 2026-09-25

**Authors:** Prashikkumar Bagade, Bikash Jyoti Borthakur, Ganesan S, Swathika B

**Affiliations:** 1 Department of Conservative Dentistry and Endodontics, Mahatma Gandhi Postgraduate Institute of Dental Sciences, Puducherry, IND

**Keywords:** bioceramic sealers, cell viability and cytotoxicity, l929 fibroblasts, mtt assay, root canal sealers

## Abstract

Background: Calcium silicate-based bioceramic sealers have become increasingly popular due to their biocompatibility, bioactivity, and potential for periapical tissue integration. However, evaluating their cytotoxic effects on fibroblast cell lines remains limited.

Aim: The aim of this study was to compare the cytotoxic effects of two commercially available calcium silicate-based bioceramic root canal sealers on L929 mouse fibroblast cells, using the MTT (3-(4,5-dimethylthiazol-2-yl)-2,5-diphenyltetrazolium bromide) assay to assess cell viability following exposure to serial dilutions (100%-6.25%) of sealer extracts.

Materials and methods: Leachates were prepared by incubating for 24 hours in 2 mL of serum-free Dulbecco's Modified Eagle Medium (DMEM) through 0.2 μm polyethersulfone (PES) syringe filters from sets of specimens from both sealers. L929 mouse fibroblast cells were seeded at a density of 1×104 cells/well and treated with five serial dilutions (100%, 50%, 25%, 12.5%, and 6.25%) of sealer extracts. Cell viability was determined spectrophotometrically at 570 nm by MTT assay. Dimethyl sulfoxide (DMSO) (10%) was used as a positive control, and cells without treatment were used as a negative control.

Results: Cell viability was significantly decreased with both 100% and 50% concentrations of Bio-C Sealer (Angelus Indústria de Produtos Odontológicos S/A, Londrina, Brazil) (4.89% and 9.31%, respectively) and Prime MTA Bioceramic Root Canal Sealer (Prime Dental Products Pvt Ltd, Maharashtra, India) (5.28% and 8.30%, respectively). However, at lower concentrations, cell viability recovered significantly and approached the control level at 6.25%. At 25% concentration, a statistically significant difference (p=0.002) was observed, with Bio-C Sealer showing better cytocompatibility (92.86% vs 68.35%).

Conclusion: Bio-C Sealer and Prime MTA Bioceramic demonstrated significant cytotoxicity at high extract concentrations with acceptable biocompatibility at clinically relevant dilute concentrations. Bio-C Sealer exhibited superior cytocompatibility at the 25% concentration and a higher IC₅₀ value, indicating better overall cytocompatibility. The comparatively favourable cytocompatibility profile of Bio‑C Sealer at intermediate concentrations suggests that material selection may be influenced by its reduced cytotoxic potential, particularly in scenarios where periapical tissue contact is likely. These findings highlight the importance of considering biological performance alongside physicochemical properties when choosing endodontic sealers.

## Introduction

The primary objective of root canal treatment is complete elimination of microorganisms from the root canal system, followed by three-dimensional obturation that prevents reinfection and supports periapical healing [1]. In this procedure, root canal sealers are essential for filling the space between the obturating material and the canal walls, sealing lateral canals, and occupying anatomical irregularities that gutta-percha alone cannot reach. Sealers are meant to entomb residual bacteria, prevent nutrient leakage, and, ideally, possess favorable biological properties [2].

The majority of endodontic root canal sealers are known to have toxic properties or to exhibit varying degrees of cytotoxicity in tissues, which can cause inflammation, bone resorption, and prolonged wound healing. Preferably, root canal sealers should have acceptable biological and physicochemical properties, along with biocompatibility [3]. The flow characteristics of sealers may inadvertently push the material into the periapical tissues. The substances released by degrading sealers may remain in long-term contact with periapical tissues, thereby inducing cytotoxicity and causing irritation, inflammation, and possibly delayed healing after endodontic procedures [4,5].

Traditional root canal sealers based on zinc oxide-eugenol, epoxy resin, and calcium hydroxide have been extensively studied, but each carries biological limitations. Zinc oxide-eugenol sealers are cytotoxic due to the release of eugenol; epoxy resin-based sealers, such as AH Plus (Dentsply Sirona Inc., Charlotte, North Carolina, United States), are also mechanically strong but can cause an inflammatory response when components leach into periapical tissues. This led to the development of a new generation of calcium silicate-based bioceramic sealers [6,7].

In the last few years, calcium silicate sealers have revolutionized endodontic obturation. In the presence of moisture, hydration occurs, triggering a setting reaction that produces calcium hydroxide and calcium silicate hydrate, creating an alkaline environment that promotes healing of periapical tissues [8]. In recent years, the use of bioceramic-based endodontic sealers has increased in popularity due to their biocompatibility, stability, low postoperative pain, and ability to induce biomineralization. Bioceramic sealers are generally formulated from zirconium oxide, calcium silicates, calcium phosphate monobasic, calcium hydroxide, and other fillers and thickeners [2].

Bio-C Sealer (Angelus Indústria de Produtos Odontológicos S/A, Londrina, Brazil) is a ready-mixed, resin-free calcium silicate-based sealer. The viscosity is based on polyethylene glycol, which provides excellent biocompatibility and easy removal. It contains calcium silicate, calcium hydroxide, zirconium oxide, and polyethylene glycol and has an approximate setting time of 120 minutes in the presence of humidity [8].

Another premixed, ready-to-use silicate-based sealer composed of fine, hydrophilic particles with sufficient radiopacity is the Prime MTA Bioceramic Root Canal Sealer (Prime Dental Products Pvt Ltd, Maharashtra, India). It sets hard in the presence of moisture, with a setting time between 10 and 24 hours, and exhibits the class properties of tricalcium silicate materials, releasing calcium and hydroxyl ions during hydration.

Despite the growing literature on bioceramic sealers, direct comparative evaluations of cytotoxicity among newer-generation premixed formulations, such as Bio-C Sealer and Prime MTA Bioceramic, remain limited. Freshly mixed sealers may unintentionally penetrate the root apex and come into contact with nearby tissues for a few minutes to hours prior to hardening [9]. As most biocompatibility and toxicity experiments on endodontic sealers use diluted extracts from preconfigured sealers, understanding the cytotoxic profiles across a range of concentrations is essential for meaningful biocompatibility assessment.

Therefore, using a standardized MTT (3-[4,5-dimethylthiazol-2-yl]-2,5-diphenyltetrazolium bromide) cell viability assay on L929 mouse fibroblasts, the present in vitro study was designed to assess and compare the cytotoxic effects of two commercially available bioceramic sealers (Bio-C Sealer and Prime MTA Bioceramic Root Canal Sealer).

## Materials and methods

This was an in vitro comparative study conducted at the Pondicherry Center for Biological Science and Educational Trust (PCBSET), Puducherry, India, from January 2026 to April 2026, to evaluate and compare the cytotoxic effects of two calcium silicate-based bioceramic root canal sealers: Bio-C Sealer and Prime MTA Bioceramic Root Canal Sealer. A total of five test concentrations (100% (undiluted), 50%, 25%, 12.5%, and 6.25%) were assessed for each sealer in triplicate, along with a negative control (untreated cells) and a positive control (10% dimethyl sulfoxide (DMSO)), yielding 21 experimental wells per sealer. Cytotoxicity was evaluated in L929 mouse fibroblasts using the MTT assay. The L929 line is a well-characterized murine fibroblast line recommended by ISO 10993-5 for cytotoxicity testing of medical device materials, and the sample surface area to extraction medium ratio (sealer spread across the full surface of a 12-well plate well, extracted in 2mL Dulbecco’s Modified Eagle Medium (DMEM)) was kept constant in both sealers.

Leachate preparation

Sealer extracts were prepared following a standardized protocol based on manufacturer instructions. Each sealer was placed in an individual well of a sterile 12-well culture plate, and the material was spread to ensure it covered the entire well surface. Plates were incubated for 24 hours at 37°C to allow full setting. The specimens were then placed under ultraviolet (UV) radiation for 30 minutes on each side to obtain sterility within a laminar airflow cabinet, ensuring aseptic handling of the sealer discs before extract preparation; subsequent 0.2 μm polyethersulfone (PES) filtration of the leachate provided an additional sterility safeguard.

After sterilization, 2 mL of antibiotic/anti-mycotic-free and serum-free DMEM was added to each well. The sealer extracts were then obtained by incubating the plates for 24 hours at 37°C, 95% humidity, and 5% CO2. The resulting leachates were filtered using PES syringe filters (0.2 μm; sterile; Cat No: PN4612; Pall Corporation, Port Washington, New York, United States) and stored at 4°C until further use [10,11].

Cell culture and MTT assay

The test cell line used was the murine fibroblast cell line L929. DMEM supplemented with 1X antibiotic-antimycotic solution and 10% fetal bovine serum (HiMedia Laboratories Private Limited, Thane, Maharashtra, India) was used to plate cells at a concentration of 1×10^4^ cells/well in 96-well plates. Cultures were maintained in a CO2 incubator at 37 °C and 5% CO2.

We used 0.2 μm syringe filters to filter-sterilize the sealer extracts. Cells were cleaned with 200 μL of 1X phosphate-buffered saline (PBS) and subsequently treated with five serial dilutions of each sealer extract (100%, 50%, 25%, 12.5%, and 6.25% v/v) prepared in serum-free medium. The negative control was untreated cells, and the positive control was DMSO (10%). The test samples were incubated with the cells for 24 hours.

After the treatment period ended, the medium was aspirated, and each well received 0.5 mg/mL MTT dissolved in 1X PBS. In a CO2 incubator, plates were kept at 37°C for four hours. After discarding the MTT-containing medium, cells were washed with 200 μL PBS, and formazan crystals were thoroughly mixed and dissolved in 100 μL of DMSO. A microplate reader was used to measure the absorbance at 570 nm. The percentage of cell viability in relation to the negative control group was reported. The dose-response curve was fitted to determine the IC_50_ [10,11].

Statistical analysis

Data analysis was conducted using IBM SPSS Statistics for Windows, version 26.0 (IBM Corp., Armonk, New York, United States). Within each sealer group, the effect of concentration on cell viability was evaluated using repeated-measures ANOVA. When overall ANOVA results were significant, pairwise comparisons were carried out using Tukey's post hoc test. An independent-samples t-test was used to compare cell viability among the sealers at each concentration. A p-value of less than 0.05 was considered statistically significant. IC_50_ values were determined by nonlinear regression analysis.

## Results

Bio-C Sealer exhibited a clear dose-dependent cytotoxic response, with cell viability below 10% at 100% and 50% extract concentrations. Marked recovery was observed at 25% concentration; viability approached control levels, and this trend continued at 12.5% and 6.25%, with viability comparable to the negative control (Figure 1). The IC₅₀ was calculated as 36.09 ± 2.91%.

**Figure 1:**
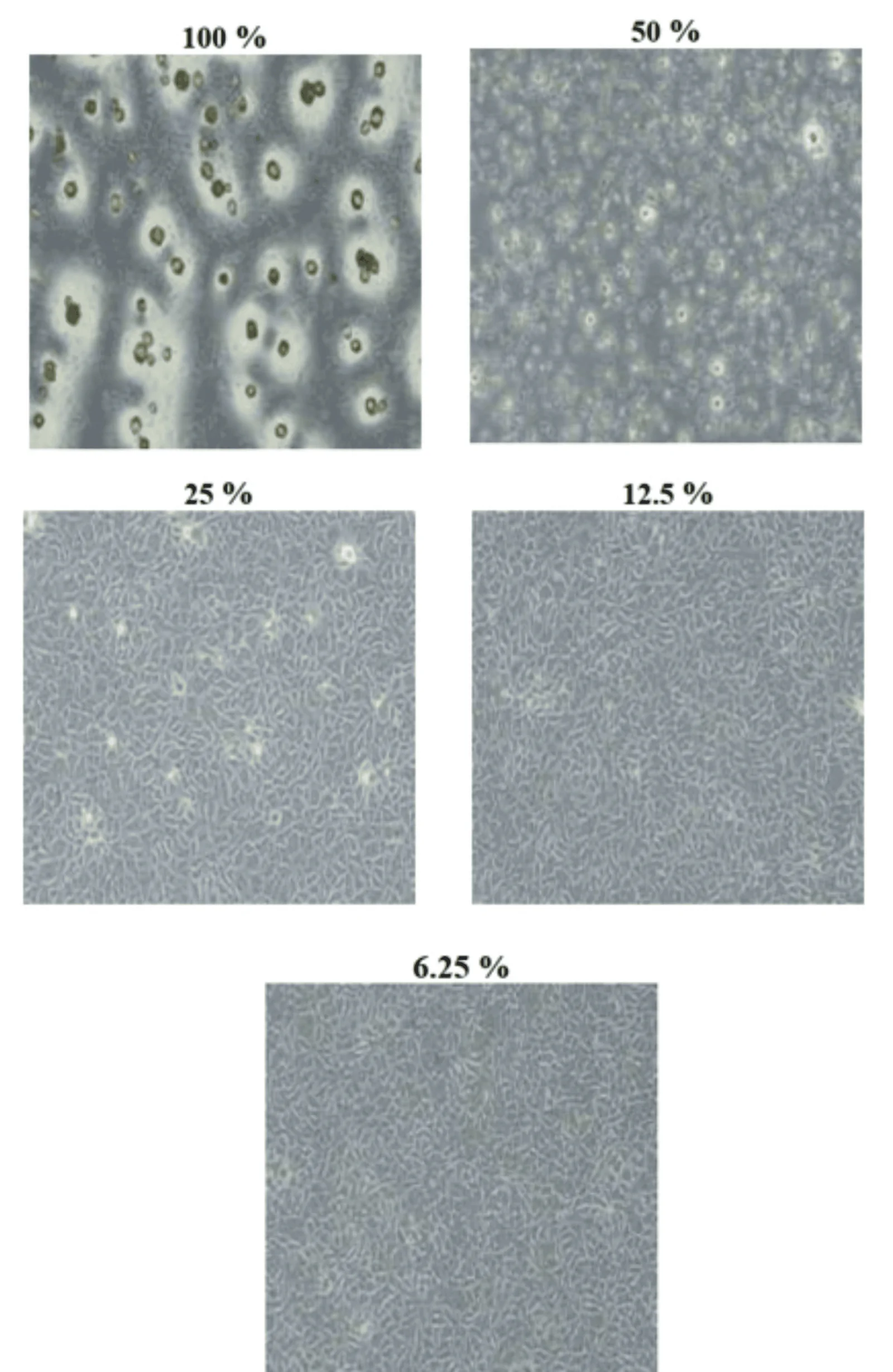
Microscopic image of L929 fibroblast cells treated with Bio-C Sealer* extract at different concentrations (100%, 50%, 25%, 12.5%, and 6.25%) Significant loss of viability was shown at 100% and 50% concentrations, with sparse clusters and altered morphology. Partial recovery of cell density is observed at 25% concentration. Morphologically intact cells with lower cytotoxic effect are observed at 12.5% and 6.25%. Images were captured using phase-contrast inverted microscopy (Model IM-3FL4; Optika SRL, Ponteranica, Italy) fitted with a P20CC Pro Cooled Color camera (20 MP CMOS), at 100× total magnification (10× objective × 10× eyepiece). *Angelus Indústria de Produtos Odontológicos S/A, Londrina, Brazil

Pronounced cytotoxicity was also observed for Prime MTA Bioceramic Sealer at undiluted and 50% concentrations. However, the recovery at 25% dilution was not as complete as that of Bio-C Sealer, and viability at this concentration remained significantly below that of the controls. Near-control viability was restored only at 12.5% and 6.25% dilutions (Figure 2). The IC₅₀ was 29.66 ± 1.82%, which was lower than that of Bio-C Sealer, indicating comparatively greater cytotoxicity.

**Figure 2:**
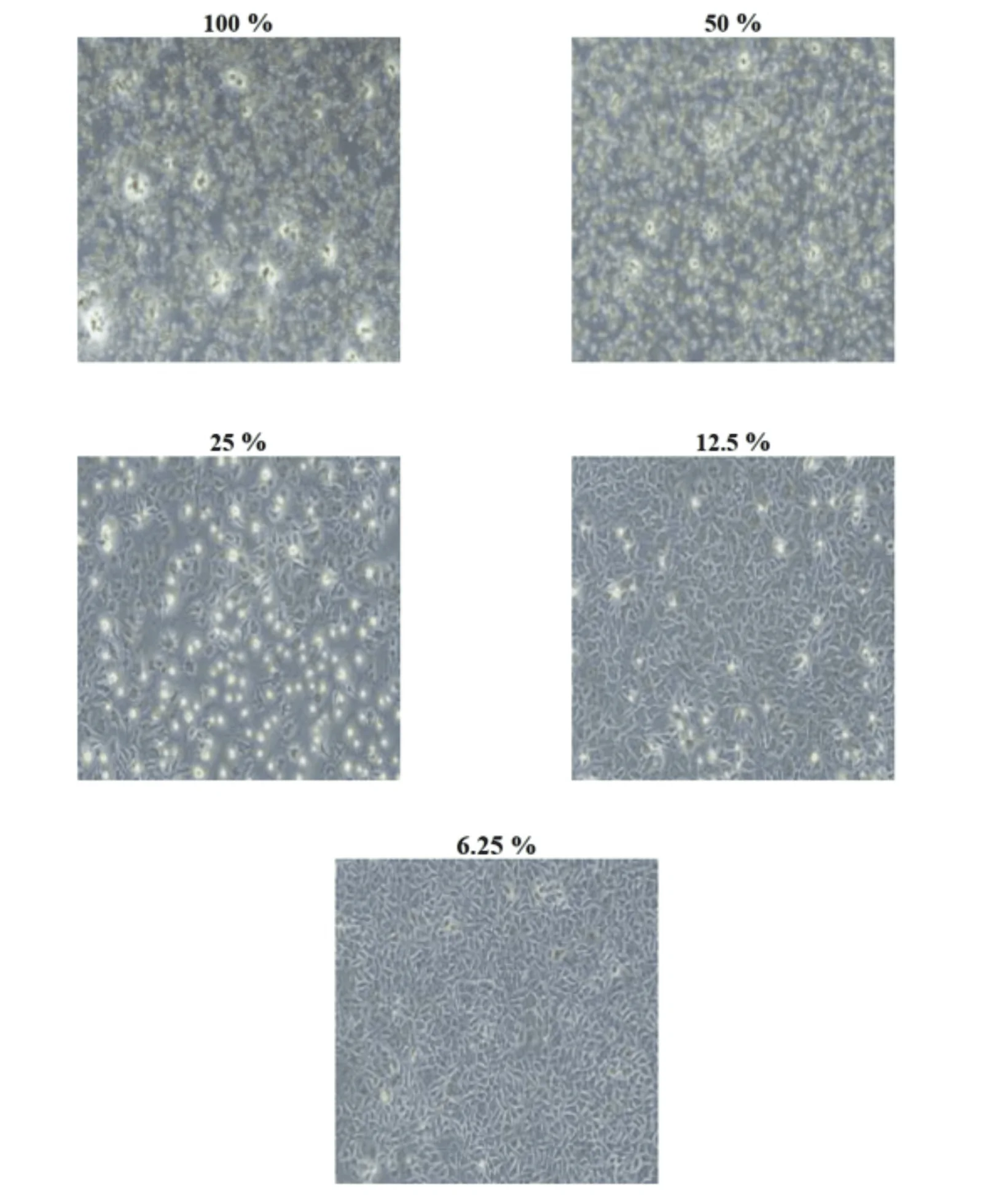
Microscopic images of L929 fibroblast cells after exposure to Prime MTA Bioceramic Root Canal Sealer* extract at different concentrations (100%, 50%, 25%, 12.5% and 6.25%). High concentrations (100% and 50%) showed pronounced cytotoxicity and reduced viable cell density. An intermediate concentration (25%) displays moderate viability, and lower concentrations (12.5% and 6.25%) show normal fibroblast morphology. Images were captured using phase-contrast inverted microscopy (Model IM-3FL4; Optika SRL, Ponteranica, Italy) fitted with a P20CC Pro Cooled Color camera (20 MP CMOS), at 100× total magnification (10× objective × 10× eyepiece). *Prime Dental Products Pvt Ltd, Maharashtra, India

Bio-C Sealer showed a higher IC_50_ value than Prime MTA Sealer, meaning that more Bio-C Sealer is required to reduce cell viability to 50%, indicating better cytocompatibility.

Within-group comparison confirmed a highly significant dose-dependent effect of concentration on cell viability for both materials (p < 0.001), demonstrating that increasing dilution consistently improved cytocompatibility for both sealers. Intergroup comparison revealed that the two sealers performed comparably across most concentrations (p > 0.05), with the exception of the 25% concentration, where Bio-C Sealer demonstrated significantly superior cell viability (t = 6.79, p = 0.002) (Table 1). 

**Table 1:** Comparison of cell viability for Bio-C Sealer** and Prime MTA Bioceramic Sealer*** *Statistically significant (p < 0.05); ^a^ Independent t test; ^b ^Repeated Measures ANOVA Conc.: concentration; SE: standard error **Angelus Indústria de Produtos Odontológicos S/A, Londrina, Brazil; ***Prime Dental Products Pvt Ltd, Maharashtra, India

Concentration (%)	Bio-C sealer	Prime MTA	Mean difference	SE difference	t	95% CI (Lower)	95% CI (Upper)	p value^a^
100	4.89±0.62	5.28±1.25	-0.39	0.80	-0.49	-2.11	1.33	0.65
50	9.31±1.65	8.30±1.27	1.01	1.22	0.83	-1.60	3.62	0.44
25	92.86±5.11	68.35±3.57	24.51	3.61	6.79	16.75	32.27	0.002*
12.5	97.05±6.41	90.77±4.48	6.28	4.45	1.41	-3.29	15.85	0.23
6.25	99.61±3.89	99.30±2.23	0.31	2.58	0.12	-5.24	5.86	0.91
F	742.15	681.32						
p value^b^	< .001*	< .001*						

Pairwise comparison of cell viability within the sealer group is presented in Table 2. 

**Table 2:** Pairwise comparisons of cell viability within each sealer group using Tukey's post hoc test *Statistically significant (p < 0.05)

Group	Comparison	Mean difference	p value
Bio-C Sealer (Angelus Indústria de Produtos Odontológicos S/A, Londrina, Brazil)	100 vs 50	-4.42	0.041*
	100 vs 25	-87.97	< .001*
	100 vs 12.5	-92.16	< .001*
	100 vs 6.25	-94.72	< .001*
	50 vs 25	-83.55	< .001*
	50 vs 12.5	-87.74	< .001*
	50 vs 6.25	-90.30	< .001*
	25 vs 12.5	-4.19	0.058
	25 vs 6.25	-6.75	0.012*
	12.5 vs 6.25	-2.56	0.321
Prime MTA Bioceramic Root Canal Sealer (Prime Dental Products Pvt Ltd, Maharashtra, India)	100 vs 50	-3.02	0.089
	100 vs 25	-63.07	< .001*
	100 vs 12.5	-85.49	< .001*
	100 vs 6.25	-94.02	< .001*
	50 vs 25	-60.05	< .001*
	50 vs 12.5	-82.47	< .001*
	50 vs 6.25	-91.00	< .001*
	25 vs 12.5	-22.42	< .001*
	25 vs 6.25	-30.95	< .001*
	12.5 vs 6.25	-8.53	0.006*

## Discussion

The present in vitro study compared two calcium silicate-based premixed bioceramic root canal sealers, Bio-C Sealer and Prime MTA Bioceramic, with respect to their cytotoxic effect on L929 mouse fibroblasts using the MTT assay, a well-validated method recommended by ISO 10993-5 for evaluating the cell viability of biomaterials. According to the guidelines, cell viability percentages exceeding 80% indicate non-cytotoxicity, while viability rates in the range of 80%-60% are considered weak [12]. The MTT assay measures the mitochondrial enzymatic reduction of MTT to purple formazan crystals, which is directly proportional to metabolically active cell numbers, making it a sensitive and reliable surrogate for cellular viability.

Both sealers demonstrated significant cytotoxicity at undiluted and 50% concentrations, with cell viability below 10% for both materials. As reported by Özdemir and Kopac in their review of root canal sealer cytotoxicity, most endodontic root canal sealers exhibit varying levels of cytotoxicity against tissues, and their extracts exert time- and dose-dependent effects on cells [3]. The present study found that the cytotoxic response pattern for both sealers is dose-dependent, which is entirely consistent with these established observations.

The recovery of cell viability at the 25% dilution, particularly for Bio-C Sealer (92.86%), is clinically relevant. In the periapical environment, the effective concentration of leached components that reach periapical cells is substantially diluted by tissue fluid, the blood supply, and the lymphatic network. This physiological buffering effect supports the interpretation that at clinically representative concentrations, both sealers are broadly biocompatible. This aligns with observations by Pitzschk et al., who reported that Bio-C Sealer and EndoSequence BCSealer HiFlow (Brasseler USA, Georgetown, Georgia, United States) showed similar MTT values to the control group when diluted (1:5), and concluded that the biocompatibility of calcium silicate bioceramic sealers is acceptable [13].

It is interesting to note that at 25% concentration, there is a statistically significant difference between the two sealers. At this dilution, Bio-C Sealer maintained 92.86% cell viability, and Prime MTA Sealer maintained 68.35% cell viability. This difference can be explained by compositional factors. Bio-C Sealer has no resinous base; its viscosity is provided by polyethylene glycol, which is biologically inert. Só et al. explained the lower cytotoxicity of Sealer Plus BC (calcium silicate-based sealer) to its bioceramic components, calcium hydroxide and resin-free composition, which favors an alkaline pH, greater release of Ca2+ ions, and hydroxyapatite formation, which could favor biological activity [14]. The cytocompatibility of Bio-C Sealer at 25% dilution may also be due to its compositional similarity to these calcium silicate formulations.

This is further supported by the IC_50_ comparison: Bio-C Sealer required a higher concentration (36.09%) to reduce cell viability to half compared with Prime MTA Sealer (29.66%). The closer the IC_50_ is to the undiluted concentration, the better the cytocompatibility will be in the test range. These results are in accordance with the results obtained by López-García et al., who reported that Bio-C Sealer and TotalFill BC Sealer (FKG Dentaire, La Chaux-de-Fonds, Switzerland) showed better cytocompatibility profiles than epoxy resin-based sealers on human periodontal ligament stem cells and attributed this favorable behavior to the bioceramic composition that promotes hydroxyapatite formation [15].

Só et al. assessed both calcium silicate-based (Sealer Plus BC; MK Life, Porto Alegre, Rio Grande do Sul, Brazil) and resin-based sealers (AH Plus and MTA Fillapex (Angelus Indústria de Produtos Odontológicos S/A)) using human periodontal ligament stem cells, and concluded that Sealer Plus BC had the lowest cytotoxicity at all time points evaluated, due to its bioceramic composition: calcium hydroxide and propylene glycol, which promote an alkaline pH and increased release of calcium ions [14]. This parallels our finding that Bio-C Sealer, which is also a calcium silicate formulation with polyethylene glycol as the viscosity agent, exhibited better cytocompatibility at intermediate concentrations compared with Prime MTA Sealer.

Chen et al. evaluated the cytotoxic properties of four endodontic sealer leachates and observed high cell viability across all sealer groups, with all sealers exhibiting an acceptable level of viability [16]. In contrast to indirect contact, the cytotoxicity that results from direct contact with epoxy resin-based sealers is significantly more pronounced. This is due to the lower concentration of cytotoxic components in the leachates, resulting from their low solubility. Additionally, the indirect contact method may account for the low cytotoxicity observed by cell death assessment. This is directly relevant to the present study, in which an indirect leachate extraction method was employed, and it supports the biocompatibility profiles observed at lower concentrations.

Pitzschk et al. reported that undiluted Bio-C Sealer extracts exhibited lower cell viability than AH Plus Jet in Saos-2 osteoblast cells at 24 and 48 hours, but both showed comparable viability at a 1:5 dilution [13], consistent with the cytotoxic profile normalizing at lower concentrations observed in the present study. Additionally, their investigation verified that calcium silicate bioceramic sealers, including EndoSequence BCSealer HiFlow, EndoSequence BCSealer, and AH Plus Jet, are biocompatible [13].

When comparing the cytotoxicity of epoxy resin-based sealers (Adseal (Meta Biomed, South Korea), AH Plus) with the bioceramic sealer CeraSeal (Meta Biomed) on L929 mouse fibroblasts, Shanavaz et al. found no statistically significant differences among materials at 24 and 48 hours, with only minor variation at 72 hours, indicating that all tested materials maintained acceptable levels of biocompatibility throughout the experimental period [17]. These findings, using the same cell line as the present study (L929), reinforce the broader pattern that bioceramic sealers demonstrate acceptable biocompatibility profiles.

Kim et al. studied epoxy resin-based sealers and found that unset sealers were considerably more cytotoxic than set materials, emphasizing the importance of the setting state in cytotoxicity evaluation, and concluded that unset epoxy resin-based sealers are generally more cytotoxic and genotoxic than the set materials [9]. In the present study, sealers were allowed to set for 24 hours before leachate preparation and UV sterilization, more closely approximating the post-setting clinical scenario.

While the difference at 25% concentration achieved statistical significance, applying the ISO 10993‑5 grading criteria shows that this difference is also biologically meaningful: Bio‑C Sealer (92.86%) remained within the non‑cytotoxic range (>80%), whereas Prime MTA Bioceramic Sealer (68.35%) fell into the weak cytotoxicity range (60-80%). This indicates that at this intermediate dilution, the two sealers occupy different cytotoxicity grades rather than statistical variation alone. Furthermore, the observed variation, although statistically robust, may represent a modest biological distinction rather than a decisive difference in safety profile. Care should therefore be taken not to over‑extrapolate these in vitro findings to clinical outcomes without further validation. Moreover, the comparatively higher IC_50_ of Bio-C sealer suggests that it can tolerate greater physiological dilution before exerting cytotoxic effects, potentially reflecting a wider margin of biological safety once diluted by periapical tissue fluid and the local blood supply in vivo.

A few limitations of the present study merit acknowledgment. As an in vitro investigation, the cytotoxicity observed in this cell culture model may not directly translate into equivalent clinical periapical tissue responses, since the biological interpretation of such findings is highly dependent on factors including sealer composition, setting time, extract preparation method, tested concentration, and duration of exposure, all of which may vary between experimental protocols and the clinical setting. Moreover, this study employed a single cell line (L929 mouse fibroblasts) and a 24-hour incubation endpoint, which restricts generalizability and underscores the need for validation across additional cell types and ultimately in vivo models. In vivo conditions involve significantly greater biological complexity, including blood flow, lymphatic drainage, immune response, and the dynamic interplay of multiple cell types, all of which can modulate cytotoxic outcomes. Furthermore, genotoxicity was not evaluated in the present study and should be considered in future investigations given the established dissociation between cytotoxic and genotoxic responses reported in the literature. The leachate extraction method, while standard, does not fully replicate the complex diffusion kinetics that occur in vivo, as sealers gradually release components through the apical foramen and lateral canals over time. To confirm these results and direct the choice of clinical materials, further in vivo and long-term in vitro research employing a wider variety of cell types, genotoxicity assessment, and longer assessment endpoints is necessary.

## Conclusions

Within the limitations of this in vitro study using L929 mouse fibroblast cells, both Bio‑C Sealer and Prime MTA Bioceramic Root Canal Sealer demonstrated marked cytotoxicity at undiluted and 50% extract concentrations, with cell viability falling below 10%. At clinically relevant dilutions (12.5% and 6.25%), both materials achieved >90% viability, fulfilling ISO 10993‑5 criteria for acceptable biocompatibility. A statistically significant difference was observed at the 25% concentration, where Bio‑C Sealer maintained superior cytocompatibility compared with Prime MTA Bioceramic Sealer. Furthermore, the higher IC₅₀ value of Bio‑C Sealer relative to Prime MTA Bioceramic Sealer confirms its comparatively better cytocompatibility across the tested concentration range.

These findings highlight that while both sealers are biocompatible at clinically relevant dilutions, Bio‑C Sealer demonstrates a more favorable cytotoxicity profile at intermediate concentrations. However, these results are limited to in vitro conditions, and further in vivo validation is warranted to support clinical relevance. The statistically significant difference observed at the 25% concentration should be interpreted with caution: Bio‑C Sealer fell within the non‑cytotoxic range (>80%), whereas Prime MTA Bioceramic Sealer was classified as weakly cytotoxic (60-80%) under ISO 10993‑5 criteria. Such distinctions, while biologically meaningful in vitro, may not directly translate into clinically significant differences in periapical tissue safety.
